# Impact of Super-Resolution Deep Learning Reconstruction on Low-Dose CT in Patients with Central Venous Catheter or Central Venous Port

**DOI:** 10.1007/s10278-025-01726-w

**Published:** 2025-10-28

**Authors:** Koichiro Yasaka, Miyaka Wada, Kishin Tokuyama, Taku Takaishi, Osamu Abe

**Affiliations:** Department of Radiology, The University of Tokyo, Tokyo, Japan

**Keywords:** Super-resolution deep learning reconstruction, CT, Radiation dose, Central venous catheter, Central venous port

## Abstract

CT imaging is useful for evaluating complications related to central venous catheter insertion; however, it involves exposure to ionizing radiation. This study aimed to evaluate the impact of super-resolution deep learning reconstruction (SR-DLR) on the quality of low-dose CT imaging compared to hybrid iterative reconstruction (HIR) in patients with central venous catheter or central venous port. In this retrospective study, chest CT images were reconstructed using SR-DLR and HIR from source data acquired with a three-dimensional landmark scan during scout imaging. Three readers independently assessed the quality of images in terms of noise, artifacts, depiction of the brachiocephalic vein and superior vena cava, and ease of evaluating hematoma and catheter location. The standard deviation of CT attenuation within a region of interest placed in the right atrium was recorded as quantitative image noise. All readers rated noise, artifacts, and depiction of the brachiocephalic vein as significantly improved in SR-DLR compared to HIR (*p* ≤ 0.042). Two out of three readers rated depiction of the superior vena cava, ease of evaluating hematoma, and catheter location as significantly improved in SR-DLR compared to HIR (*p* ≤ 0.016). Quantitative image noise in SR-DLR was 10.2 Hounsfield unit, which was significantly reduced compared to HIR (24.0 Hounsfield unit) (*p* < 0.001). When sufficient information can be obtained from these images, the main CT scans may be omitted. In conclusion, SR-DLR enhanced the quality of low-dose CT imaging compared to HIR in patients with central venous catheter or central venous port.

## Introduction

Central venous catheters are widely utilized for the delivery of medications, fluids, and parenteral nutrition [1]. Central venous port devices also find widespread application in patients requiring long-term intravenous therapy [2]. While central venous catheters and central venous ports offer significant advantages, central venous catheter insertion is associated with various complications, including placement failure, arterial puncture, and pneumothorax [1].

Choosing the internal jugular vein (IJV) can reduce the risk of pneumothorax compared to the subclavian vein (1.9 vs. 7.8 events per 1000 catheters) [1]. Operator experience plays a crucial role in determining the success rate of catheterization [3]. Additionally, imaging modalities play a pivotal role in mitigating the risk of complications.

During vein puncture, the use of ultrasound is recommended [4]. Ultrasound-guided puncture reduces the risk of arterial puncture and pneumothorax compared with partial or no ultrasonography use [1]. However, ultrasound alone may not adequately assess certain complications, such as placement failure (in which catheter is not properly placed in the intended location), cervical/mediastinal hematoma, and pneumothorax. CT imaging is particularly beneficial in evaluating these complications.

CT provides a detailed visualization of structures deep within the patient’s body. Unlike ultrasound, CT images are not contingent upon the operator’s experience. However, CT is associated with radiation exposure, which carries potential cancer risks [5]. Consequently, the indications for CT examination must be carefully evaluated, considering the balance between the benefits and the risks of radiation exposure.

Since the mid-2010s, applications of deep learning have garnered significant attention in the field of radiology [6, 7]. Deep learning has been demonstrated to assist radiologists in various tasks, including lesion detection [8–10], differential diagnosis [11, 12], natural language processing [13–18], and image processing [19–26]. Super-resolution DLR (SR-DLR) is a notable example of such techniques. While radiation dose is negatively correlated with noise in CT images [27], SR-DLR, trained using low-quality data and high-quality images as input and reference data, respectively, possesses the potential to reduce image noise [28–31] and to improve the image quality of CT images scanned with reduced radiation dose. Therefore, we hypothesized that CT images scanned with reduced radiation doses and reconstructed using SR-DLR would aid less experienced radiologists in identifying the course of central venous catheter in the veins and evaluating complications associated with central venous catheter insertion. If combined with a newly developed technique that uses tomographic images as scout imaging, there is potential to omit the main scan, thereby reducing unnecessary radiation exposure. It would also be useful for pre-procedural checks when administering contrast media via a CV port for a CT scan.

The aim of this study was to evaluate the impact of SR-DLR on the quality of low-dose CT images in patients with central venous catheter or central venous port, comparing them to conventional images.

## Materials and Methods

This retrospective study was approved by our Institutional Review Board, which waived the requirement for obtaining written informed consent from patients.

### Patients

In this study, patients with central venous port or central venous catheter inserted via the right internal jugular vein who underwent CT imaging with a single scanner (Aquilion ONE INSIGHT edition, Canon Medical Systems) involving the chest region from March 2025 to May 2025 were included. Patients whose images lacked the catheter insertion site were excluded (*n* = 7).

### CT Imaging

Patients underwent scout imaging with a three-dimensional landmark scan (Canon Medical Systems) mode. In this mode, tomographic images are obtained with a helical scan mode at a radiation dose almost equivalent to conventional scout imaging. In clinical practice, CT images reconstructed from this scan data allow radiographers to locate the scan region more precisely. In this study, SR-DLR (Precise IQ Engine, commercially available from Canon Medical Systems) and conventional hybrid iterative reconstruction (HIR) images (Adaptive Iterative Dose Reduction 3D [Canon Medical Systems]) were reconstructed from the data acquired with three-dimensional landmark scan mode, and these images were used for the final analyses.

Scan parameters were the following: tube voltage, 120 kVp; tube current, 40 mA; gantry rotation time, 0.5 s; and helical pitch, 1.388. Images were reconstructed with a number of pixels of 1024 and 512 for SR-DLR and HIR, respectively. Other reconstruction parameters were kept constant among the two reconstruction algorithms and were the following: kernel, body; slice thickness/interval, 3 mm/3 mm; and field of view, 351.6 mm (adjusted to patient’s body size).

### Qualitative Image Analysis

Three readers (readers 1, 2, and 3, with imaging experience of 7 years, 2 years, and 2 months, respectively) participated in qualitative image analyses. Prior to the evaluation, a radiologist (radiologist A, with 15 years of imaging experience) randomized all image datasets. Three readers were blinded to the patient’s background information. Using ImageJ software (https://imagej.net/ij/), they independently evaluated the image datasets in the following categories:

*Noise (5 = no or minimal noise, 4 = standard noise, 3 = slightly more noise, 2 = moderately prominent noise, 1 = severe noise interfering with diagnosis).

*Artifacts (4 = no or minimal artifacts, 3 = mild artifacts, 2 = moderate artifacts, 1 = severe artifacts interfering with diagnosis).

*Depiction of intrathoracic veins (right brachiocephalic vein [BCV] and superior vena cava [SVC] were evaluated separately) (4 = clear, 3 = slightly unclear for only a small region, 2 = unclear for some regions, 1 = unclear overall).

*Ease of evaluating hematoma (4 = optimal, 3 = slight degradation in image quality but not affecting evaluation, 2 = degradation in image quality slightly affecting evaluation, 1 = unacceptable) (Please note that, since there were no patients with hematoma, it was not the depiction of hematomas but rather the ease of evaluating them that was assessed.)

*Ease of localizing the catheter (4 = optimal, 3 = slight degradation in image quality but not affecting evaluation, 2 = degradation in image quality slightly affecting evaluation, 1 = unacceptable).

### Quantitative Image Analysis

**Fig. 1:**
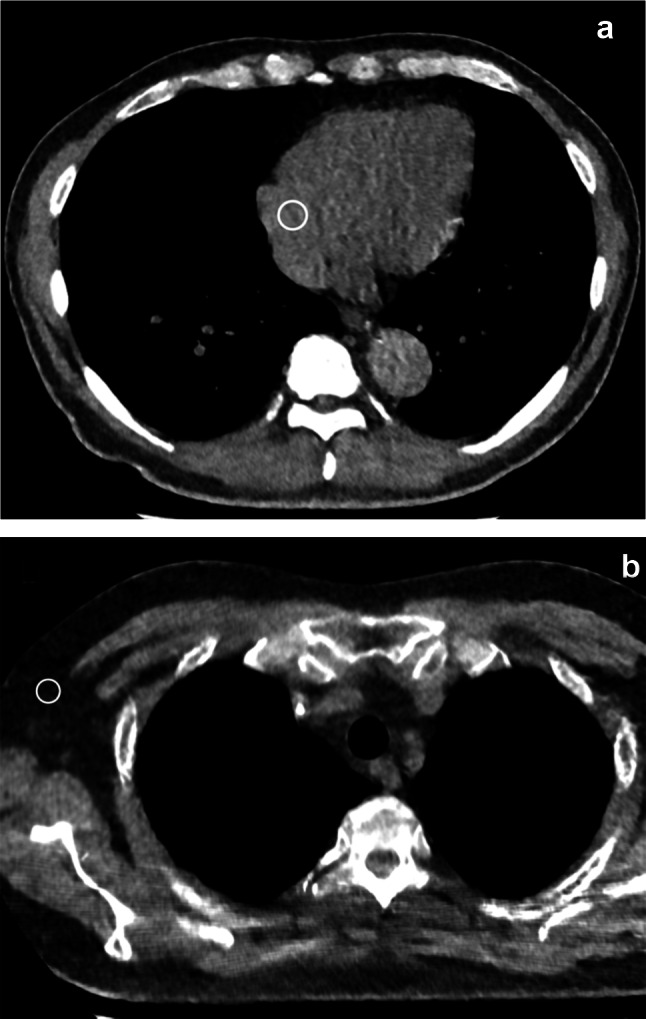
In quantitative image analyses, a circular or ovoid region of interest (white circle) was placed on the right atrium of the heart (**a**) and subcutaneous fat in the right axilla (**b**). Subsequently, the mean and standard deviation of CT attenuation within the region of interest on the right atrium and the mean CT attenuation within the region of interest on the subcutaneous fat were recorded

Radiologist A placed a circular or ovoid region of interest (ROI) with diameter of around 10 mm on the right atrium of the heart and on the subcutaneous fat in the right axilla using ImageJ software (Fig. 1). The ROI was placed on SR-DLR images and was copied to HIR images so that the location and the size are the same among them. Subsequently, the mean and standard deviation of CT attenuation within the ROI in the right atrium were recorded, with the standard deviation representing quantitative image noise. The mean of CT attenuation within the ROI in the subcutaneous fat was also recorded. Contrast-to-noise ratio (CNR) was calculated with the following formula:

$$\text{CNR }= ({\mathrm{CT}}_{\mathrm{RA}} -{\text{ CT}}_{\mathrm{FAT}})/{\mathrm{SD}}_{\mathrm{RA}}$$

where CT_RA_, CT_FAT_, and SD_RA_ denote mean CT attenuation of the right atrium, mean CT attenuation of the subcutaneous fat, and standard deviation for CT attenuation of the right atrium, respectively.

### Statistical Analysis

Statistical analyses were conducted using R version 4.1.2 (https://www.r-project.org/). Comparisons were conducted between SR-DLR and HIR for both continuous and ordinal variables using paired *t*-tests and Wilcoxon signed rank tests, respectively. A *p* value less than 0.050 was deemed statistically significant.

## Results

### Patients

In this study, 34 patients (mean age, 65.0 ± 10.7 years; 26 males and 8 females) were included. Among them, central venous port systems and central venous catheters were inserted in 29 and 5 patients, respectively. Venous puncture was performed at the right internal jugular vein for all patients. The catheter tip was located at the appropriate region (i.e., superior part of the SVC) for all patients. Indications for CT examination were the following: evaluation of colorectal cancer (*n* = 15), evaluation of pancreatic cancer (*n* = 6), evaluation of other neoplasms (*n* = 6), evaluation of focus of inflammation (*n* = 3), follow-up for acute pancreatitis (*n* = 1), follow-up for aortic dissection (*n* = 1), evaluation of nausea (*n* = 1), and evaluation of elevated liver enzyme (*n* = 1).

CT dose index volume was 0.2 mGy for all patients. Dose length product was 15.4 ± 1.4 mGy-cm for patients scanned for thoracic to pelvic regions and was 18.2 ± 2.0 mGy-cm for those scanned for neck to pelvic regions.

### Qualitative Image Analysis

**Fig. 2:**
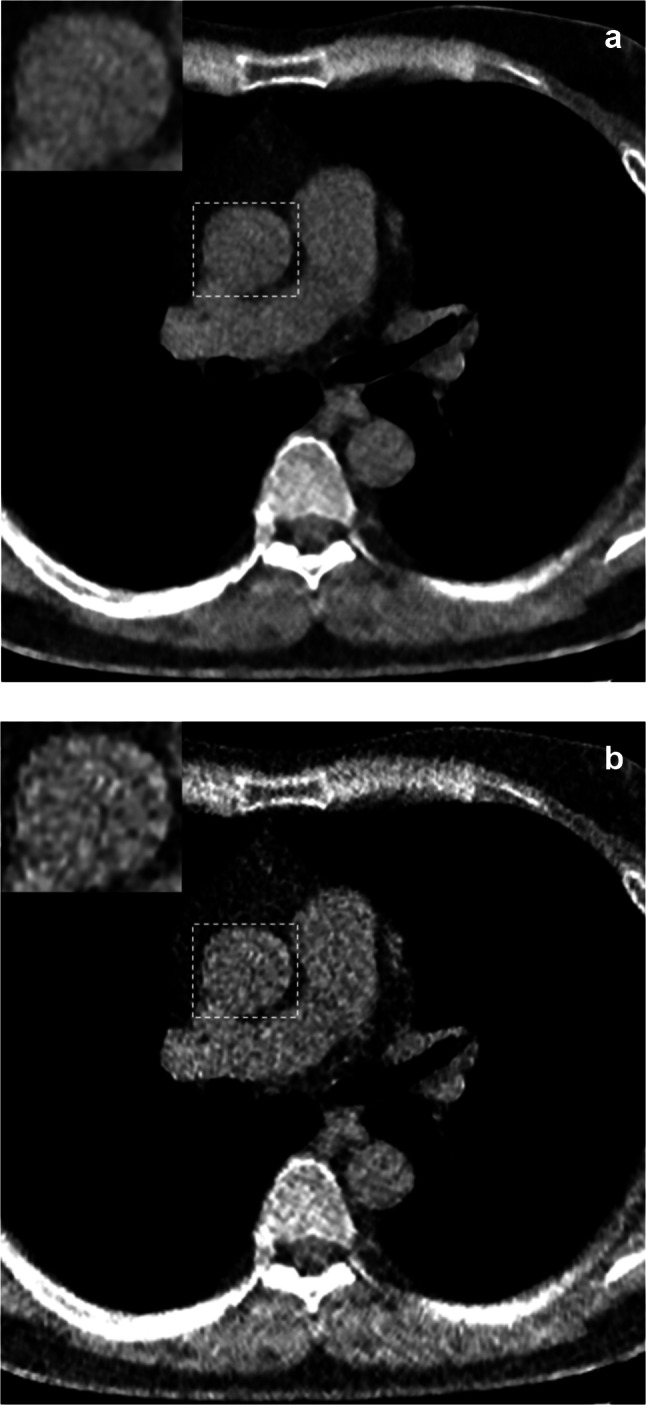
CT images ((**a**) super-resolution deep learning reconstruction [SR-DLR] and (**b**) hybrid iterative reconstruction [HIR]) of a 53-year-old male patient scanned at a radiation dose exposure nearly equivalent to scout imaging. The regions enclosed by the dotted white rectangle are magnified in the top-left inset. Image noise was rated as 3 (slightly more noise)/3/3 and 1 (severe noise interfering with diagnosis)/1/1 by readers 1/2/3 in SR-DLR and HIR, respectively

**Fig. 3:**
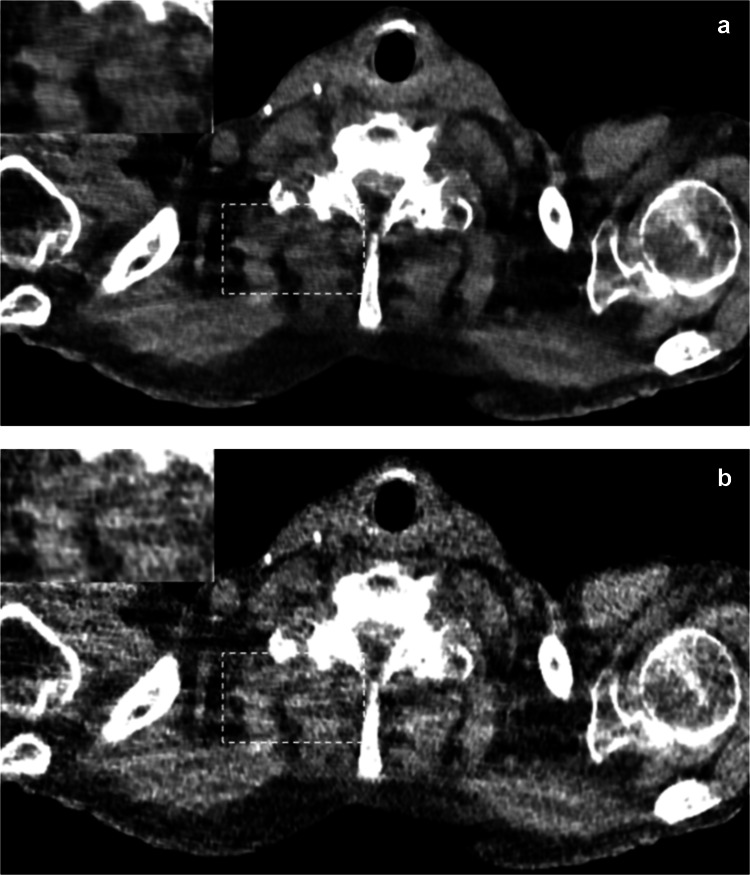
CT images ((**a**) super-resolution deep learning reconstruction [SR-DLR] and (**b**) hybrid iterative reconstruction [HIR]) of a 61-year-old male patient scanned at a radiation dose exposure nearly equivalent to scout imaging. The regions enclosed by the dotted white rectangle are magnified in the top-left inset. Artifacts were rated as 3 (mild artifacts)/3/2 (moderate artifacts) and 1 (severe artifacts interfering with diagnosis)/1/1 by readers 1/2/3 in SR-DLR and HIR, respectively

**Fig. 4:**
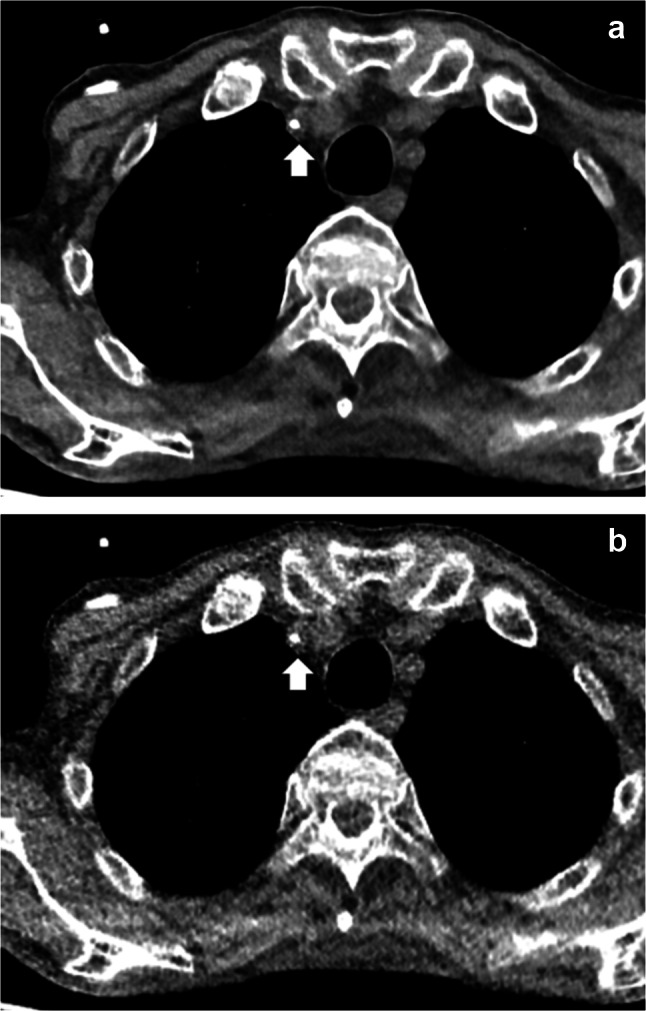
CT images ((**a**) super-resolution deep learning reconstruction [SR-DLR] and (**b**) hybrid iterative reconstruction [HIR]) of a 73-year-old male patient scanned at a radiation dose exposure nearly equivalent to scout imaging. Depiction of brachiocephalic vein (arrows) was rated as 3 (slightly unclear for only a small region)/3/4 (clear) and 1 (unclear)/2 (unclear for some regions)/4 by readers 1/2/3 in SR-DLR and HIR, respectively

Results for qualitative image analyses are detailed in Table 1. All readers agreed that noise (Fig. 2), artifact (Fig. 3), and depiction of the brachiocephalic vein (Fig. 4) in SR-DLR were significantly better than HIR (*p* ≤ 0.042). Regarding the depiction of the superior vena cava, ease of evaluating hematoma, and ease of localizing the catheter, readers 1 and 2 rated SR-DLR as superior to HIR (*p* ≤ 0.016); however, there were no statistically significant differences in scores for these evaluation items for reader 3 (*p* ≥ 0.071).

**Table 1:** Qualitative image analysis results

	Reader	SR-DLR	HIR	Comparisons
Noise	1	0/24/10/0	0/0/10/24	< 0.001*
	2	1/29/3/1	0/0/5/29	< 0.001*
	3	4/25/3/2	0/2/22/10	< 0.001*
Artifact	1	0/11/20/3	0/1/13/20	< 0.001*
	2	0/1/23/10	0/0/16/18	0.023*
	3	0/5/22/7	0/3/14/17	0.008*
Depiction of the brachiocephalic vein	1	0/22/9/3	0/13/16/5	0.042*
	2	0/21/12/1	1/10/19/4	0.015*
	3	30/1/2/1	23/6/5/0	0.040*
Depiction of the superior vena cava	1	1/31/2/0	0/24/10/0	< 0.001*
	2	13/19/2/0	5/24/4/1	0.016*
	3	30/3/1/0	26/5/3/0	0.071
Evaluation of hematoma	1	0/26/8/0	0/7/27/0	< 0.001*
	2	0/1/25/8	0/0/7/27	< 0.001*
	3	11/17/6/0	18/10/4/2	0.340
Evaluation of catheter location	1	0/18/15/1	0/6/26/2	< 0.001*
	2	30/4/0/0	20/13/1/0	0.013*
	3	17/14/3/0	18/11/3/2	0.458

The numbers of patients for each score (4/3/2/1) are presented. Comparisons between SR-DLR vs. HIR were performed with the Wilcoxon signed rank test

*SR-DLR* super-resolution deep learning reconstruction, *HIR* hybrid iterative reconstruction

^*^Statistically significant difference (*p* < 0.050)

### Quantitative Image Analysis

Mean CT attenuation of the right atrium of the heart in SR-DLR and HIR was 49.8 ± 10.2 and 50.8 ± 10.4 Hounsfield unit (HU), respectively. There was no statistically significant difference between them (*p* = 0.053, power = 0.079). Quantitative image noise in SR-DLR and HIR was 10.2 ± 1.4 and 24.0 ± 2.8 HU, respectively, and there was a statistically significant difference between them (*p* < 0.001). Mean CT attenuation of the subcutaneous fat in SR-DLR (− 80.6 ± 11.4 HU) was significantly higher than that in HIR (− 82.6 ± 12.2 HU) (*p* = 0.004). CNR in SR-DLR (13.0 ± 2.5) was also significantly higher than that in HIR (5.6 ± 1.1) (*p* < 0.001).

## Discussion

CT images are instrumental in diagnosing complications associated with central venous catheter insertion. However, CT is associated with relatively high radiation exposure. Reducing radiation dose can degrade image quality. In this study, we found that SR-DLR was effective in enhancing the quality of low-dose CT images and in better visualizing the course of central venous catheter compared to conventional images reconstructed with HIR algorithm for the majority of readers. Since we used the commercially available precise IQ engine as the SR-DLR algorithm, we believe our study results are directly applicable to CT images reconstructed with the same algorithm.

Central venous catheters and central venous ports are widely used in daily clinical practice. However, the insertion of central venous catheter is not without complications, such as mispositioning of the catheter (into the azygos vein, internal mammary vein, left superior intercostal vein, etc.), cervical/mediastinal hematoma, and pneumothorax [2]. On single-plane chest radiographs, placement into the right internal mammary vein can be challenging to detect [2]. Conversely, CT is a useful modality in identifying these complications. It has been reported that radiation exposure from CT poses potential cancer risks [5]. However, reducing radiation dose can compromise the quality of CT images. A novel three-dimensional landmark scan technique is an idea to obtain scout images as tomographic images with helical scan mode. In this technique, radiation exposure is virtually equivalent to that in conventional scout imaging. In our study, we applied SR-DLR technique to a three-dimensional landmark scan to compensate for degraded quality. Currently, SR-DLR algorithm is commercially available only from Canon Medical Systems as PIQE [32].

Image noise reduction was demonstrated through both quantitative and qualitative analyses. Quantitative image noise was significantly reduced compared to HIR (from 24.0 to 10.2 HU) (*p* < 0.001). Additionally, all readers rated that image noise in SR-DLR as significantly reduced compared to HIR (*p* < 0.001). This would have resulted in an increased CNR for SR-DLR compared to HIR. The potential of SR-DLR in reducing image noise compared with conventional reconstruction algorithms at standard radiation dose levels has been demonstrated in previous studies [28–31]. Although the radiation dose used in CT scanning differs from that in those studies, our results are consistent with theirs. Photon starvation is also associated with streak artifacts [33–35]. In our study, artifacts in SR-DLR were rated as significantly reduced compared to HIR by all readers (*p* ≤ 0.023). Image quality enhancement, as evidenced by these indicators, would have resulted in improved depiction of the brachiocephalic vein by all readers (*p* ≤ 0.042) and depiction of the superior vena cava and ease of evaluation of hematoma and catheter location by two out of three readers (*p* ≤ 0.016). The discrepancy in the results for the latter evaluation items may have been attributed to the reader’s diagnostic imaging experience, as the results for readers with relatively longer experience were consistent for those evaluation items. In patients planning to undergo a CT examination to evaluate complications associated with the insertion of central venous catheter or central venous port, if three-dimensional landmark scan images reconstructed with SR-DLR possess adequate quality to evaluate those complications, the main scan may be omitted, thereby reducing unnecessary radiation exposure to patients. The mean CT dose index volume in our study was 0.2 mGy, which was 80 times below the national diagnostic reference levels in Japan (16 mGy for chest to pelvis) (https://j-rime.qst.go.jp/report/DRL2020_Engver.pdf).

There are some limitations with this study. Firstly, the retrospective design of the study may introduce selection bias. However, the promising results observed warrant the conduct of a prospective study with a larger participant cohort. Secondly, there were no patients who experienced complications after catheter insertion or mispositioned central venous catheter. Future studies need to investigate the diagnostic performance for complications or mispositioned central venous catheter. Thirdly, we did not compare the quality of low-dose CT images with that of routine-dose CT images. Because the quality of the latter images is better than the former images from our preliminary observation. Therefore, we propose omitting the main CT scan only when the scout imaging reconstructed with SR-DLR has enough quality for the diagnosis of complications. Fourthly, the number of patients included in this study was relatively small, which may have affected the results regarding the difference in mean CT attenuation of the right atrium. Lastly, while similar reconstruction algorithms are available from other vendors, their detailed mechanisms differ. Therefore, the results of this study cannot be directly applied to CT data obtained with scanners from other vendors.

In conclusion, SR-DLR enhanced the quality of CT images scanned with radiation dose comparable to scout imaging compared to HIR in patients with central venous catheter or central venous port. When catheter localization or complications diagnosis is possible using these images, the main CT scan can be omitted, thereby reducing unnecessary radiation exposure to patients. It would also be useful for pre-procedural checks when administering contrast media via a CV port for a CT scan. Given the widespread use of central venous catheter and central venous port in clinical practice, our findings have the potential to significantly benefit a substantial number of patients.

## Data Availability

The datasets generated and/or analyzed during the current study are not publicly available due to paients’ confidentiality.

## References

[1] Teja B, Bosch NA, Diep C et al (2024) Complication Rates of Central Venous Catheters: A Systematic Review and Meta-Analysis. JAMA Intern Med 184(5):474-482. 10.1001/jamainternmed.2023.8232. 38436976 10.1001/jamainternmed.2023.8232 PMC12285596

[2] Machat S, Eisenhuber E, Pfarl G et al (2019) Complications of central venous port systems: a pictorial review. Insights Imaging 10(1):86. 10.1186/s13244-019-0770-2. 31463643 10.1186/s13244-019-0770-2 PMC6713776

[3] Yoon HK, Hur M, Cho H et al (2021) Effects of practitioner's experience on the clinical performance of ultrasound-guided central venous catheterization: a randomized trial. Sci Rep 11(1):6726. 10.1038/s41598-021-86322-y. 33762662 10.1038/s41598-021-86322-y PMC7991409

[4] Saugel B, Scheeren TWL, Teboul JL (2017) Ultrasound-guided central venous catheter placement: a structured review and recommendations for clinical practice. Crit Care 21(1):225. 10.1186/s13054-017-1814-y. 28844205 10.1186/s13054-017-1814-y PMC5572160

[5] Smith-Bindman R, Chu PW, Azman Firdaus H et al (2025) Projected Lifetime Cancer Risks From Current Computed Tomography Imaging. JAMA Intern Med 185(6):710-719. 10.1001/jamainternmed.2025.0505. 40227719 10.1001/jamainternmed.2025.0505 PMC11997853

[6] Yasaka K, Akai H, Kunimatsu A, Kiryu S, Abe O (2018) Deep learning with convolutional neural network in radiology. Jpn J Radiol 36(4):257-272. 10.1007/s11604-018-0726-3. 29498017 10.1007/s11604-018-0726-3

[7] Chartrand G, Cheng PM, Vorontsov E et al (2017) Deep Learning: A Primer for Radiologists. Radiographics 37(7):2113-2131. 10.1148/rg.2017170077. 29131760 10.1148/rg.2017170077

[8] Yasaka K, Hatano S, Mizuki M et al (2023) Effects of deep learning on radiologists' and radiology residents’ performance in identifying esophageal cancer on CT. Br J Radiol 96(1150):20220685. 10.1259/bjr.20220685. 37000686 10.1259/bjr.20220685 PMC10546446

[9] Ueda D, Yamamoto A, Nishimori M et al (2019) Deep Learning for MR Angiography: Automated Detection of Cerebral Aneurysms. Radiology 290(1):187-194. 10.1148/radiol.2018180901. 30351253 10.1148/radiol.2018180901

[10] Yasaka K, Sato C, Hirakawa H et al (2024) Impact of deep learning on radiologists and radiology residents in detecting breast cancer on CT: a cross-vendor test study. Clin Radiol 79(1):e41-e47. 10.1016/j.crad.2023.09.022. 37872026 10.1016/j.crad.2023.09.022

[11] Li Y, Zhang Y, Zhang E et al (2021) Differential diagnosis of benign and malignant vertebral fracture on CT using deep learning. Eur Radiol 31(12):9612-9619. 10.1007/s00330-021-08014-5. 33993335 10.1007/s00330-021-08014-5 PMC8594282

[12] Yasaka K, Akai H, Abe O, Kiryu S (2018) Deep Learning with Convolutional Neural Network for Differentiation of Liver Masses at Dynamic Contrast-enhanced CT: A Preliminary Study. Radiology 286(3):887-896. 10.1148/radiol.2017170706. 29059036 10.1148/radiol.2017170706

[13] Kanzawa J, Yasaka K, Fujita N, Fujiwara S, Abe O (2024) Automated classification of brain MRI reports using fine-tuned large language models. Neuroradiology 10.1007/s00234-024-03427-7. 38995393 10.1007/s00234-024-03427-7 PMC11611921

[14] Yasaka K, Abe O (2025) A New Step Forward in the Extraction of Appropriate Radiology Reports. Radiology 315(1):e250867. 10.1148/radiol.250867. 40232144 10.1148/radiol.250867

[15] Kanemaru N, Yasaka K, Okimoto N et al (2025) Efficacy of Fine-Tuned Large Language Model in CT Protocol Assignment as Clinical Decision-Supporting System. J Imaging Inform Med 10.1007/s10278-025-01433-6. 39909993 10.1007/s10278-025-01433-6 PMC12701208

[16] Fujita N, Yasaka K, Kiryu S, Abe O (2025) Fine-tuned large Language model for extracting newly identified acute brain infarcts based on computed tomography or magnetic resonance imaging reports. Emerg Radiol 10.1007/s10140-025-02354-1. 40451964 10.1007/s10140-025-02354-1 PMC12328549

[17] Sato M, Yasaka K, Abe S et al (2025) Efficacy of a large language model in classifying branch-duct intraductal papillary mucinous neoplasms. Abdom Radiol (NY) 10.1007/s00261-025-05062-z. 40498341 10.1007/s00261-025-05062-z PMC12830448

[18] Yamagishi Y, Nakamura Y, Hanaoka S, Abe O (2025) Large Language Model Approach for Zero-Shot Information Extraction and Clustering of Japanese Radiology Reports: Algorithm Development and Validation. JMIR Cancer 11:e57275. 10.2196/57275. 39864093 10.2196/57275 PMC11867198

[19] Kiryu S, Akai H, Yasaka K et al (2023) Clinical Impact of Deep Learning Reconstruction in MRI. Radiographics 43(6):e220133. 10.1148/rg.220133. 37200221 10.1148/rg.220133

[20] van Stiphout JA, Driessen J, Koetzier LR et al (2022) The effect of deep learning reconstruction on abdominal CT densitometry and image quality: a systematic review and meta-analysis. Eur Radiol 32(5):2921-2929. 10.1007/s00330-021-08438-z. 34913104 10.1007/s00330-021-08438-z PMC9038933

[21] Okimoto N, Yasaka K, Kaiume M, Kanemaru N, Suzuki Y, Abe O (2023) Improving detection performance of hepatocellular carcinoma and interobserver agreement for liver imaging reporting and data system on CT using deep learning reconstruction. Abdom Radiol (NY) 48(4):1280-1289. 10.1007/s00261-023-03834-z. 36757454 10.1007/s00261-023-03834-z PMC10115733

[22] Hosoi R, Yasaka K, Mizuki M et al (2023) Deep learning reconstruction with single-energy metal artifact reduction in pelvic computed tomography for patients with metal hip prostheses. Jpn J Radiol 41(8):863-871. 10.1007/s11604-023-01402-5. 36862290 10.1007/s11604-023-01402-5 PMC10366278

[23] Okimoto N, Yasaka K, Fujita N, Watanabe Y, Kanzawa J, Abe O (2023) Deep learning reconstruction for improving the visualization of acute brain infarct on computed tomography. Neuroradiology 10.1007/s00234-023-03251-5. 37991522 10.1007/s00234-023-03251-5 PMC10761512

[24] Fujita N, Yasaka K, Watanabe Y, Okimoto N, Konishiike M, Abe O (2023) Detection of Vertebral Mass and Diagnosis of Spinal Cord Compression in Computed Tomography With Deep Learning Reconstruction: Comparison With Hybrid Iterative Reconstruction. Can Assoc Radiol J:8465371231203508. 10.1177/08465371231203508. 10.1177/08465371231203508 37795610

[25] Fujita N, Yasaka K, Katayama A, Ohtake Y, Konishiike M, Abe O (2023) Assessing the Effects of Deep Learning Reconstruction on Abdominal CT Without Arm Elevation. Can Assoc Radiol J 74(4):688-694. 10.1177/08465371231169672. 37041699 10.1177/08465371231169672

[26] Hamada A, Yasaka K, Hatano S et al (2024) Deep-Learning Reconstruction of High-Resolution CT Improves Interobserver Agreement for the Evaluation of Pulmonary Fibrosis. Can Assoc Radiol J 75(3):542-548. 10.1177/08465371241228468. 38293802 10.1177/08465371241228468

[27] Catalano C, Francone M, Ascarelli A, Mangia M, Iacucci I, Passariello R (2007) Optimizing radiation dose and image quality. Eur Radiol 17 Suppl 6:F26-32. 10.1007/s10406-007-0225-6. 18376454 10.1007/s10406-007-0225-6

[28] Nagayama Y, Emoto T, Kato Y et al (2023) Improving image quality with super-resolution deep-learning-based reconstruction in coronary CT angiography. Eur Radiol 33(12):8488-8500. 10.1007/s00330-023-09888-3. 37432405 10.1007/s00330-023-09888-3

[29] Takafuji M, Kitagawa K, Mizutani S et al (2023) Super-Resolution Deep Learning Reconstruction for Improved Image Quality of Coronary CT Angiography. Radiol Cardiothorac Imaging 5(4):e230085. 10.1148/ryct.230085. 37693207 10.1148/ryct.230085 PMC10485715

[30] Yasaka K, Tsujimoto R, Miyo R, Abe O (2025) Reducing motion artifacts in the aorta: super-resolution deep learning reconstruction with motion reduction algorithm. Jpn J Radiol 10.1007/s11604-025-01849-8. 40782239 10.1007/s11604-025-01849-8 PMC12647333

[31] Takafuji M, Kitagawa K, Mizutani S et al (2025) Super-resolution deep learning reconstruction for improved quality of myocardial CT late enhancement. Jpn J Radiol 10.1007/s11604-025-01760-2. 40072715 10.1007/s11604-025-01760-2 PMC12204928

[32] Fukui R, Harashima S, Samejima W et al (2025) Acquisition and Reconstruction Techniques for Coronary CT Angiography: Current Status and Trends over the Past Decade. Radiographics 45(7):e240083. 10.1148/rg.240083. 40504731 10.1148/rg.240083

[33] Yasaka K, Furuta T, Kubo T et al (2017) Full and hybrid iterative reconstruction to reduce artifacts in abdominal CT for patients scanned without arm elevation. Acta Radiol 58(9):1085-1093. 10.1177/0284185116684675. 28068822 10.1177/0284185116684675

[34] Seyal AR, Arslanoglu A, Abboud SF, Sahin A, Horowitz JM, Yaghmai V (2015) CT of the Abdomen with Reduced Tube Voltage in Adults: A Practical Approach. Radiographics 35(7):1922-1939. 10.1148/rg.2015150048. 26473536 10.1148/rg.2015150048

[35] Yasaka K, Maeda E, Hanaoka S, Katsura M, Sato J, Ohtomo K (2016) Single-energy metal artifact reduction for helical computed tomography of the pelvis in patients with metal hip prostheses. Jpn J Radiol 34(9):625-632. 10.1007/s11604-016-0566-y. 27400700 10.1007/s11604-016-0566-y

